# IFT140 Mutation and End-Stage Renal Disease in Mainzer-Saldino Syndrome: A Case Report

**DOI:** 10.7759/cureus.53889

**Published:** 2024-02-09

**Authors:** Sara E Marhoon, Ali H Ali, Ali Husain, Ali A Alsudan, Eman G Elshabrawy

**Affiliations:** 1 College of Medicine, Mansoura University, Mansoura, EGY; 2 Pediatrics, Maternity and Children’s Hospital, Dammam, SAU; 3 Pediatric Nephrology, Mansoura University Children Hospital, Mansoura, EGY

**Keywords:** mainzer-saldino syndrome, end-stage renal disease (esrd), nephronophthisis, pneumonia, ciliopathy, case report, ift140, intraflagellar transport protein

## Abstract

Mainzer-Saldino syndrome (MSS) or conorenal syndrome (CRS) is a rare autosomal recessive ciliopathy characterized by multiorgan affection, typically presents with a triad of nephronophthisis (NPHP), retinitis pigmentosa (RP), and cone-shaped epiphysis (CSE) with varying degrees of severity. A 20-month-old male is experiencing recurrent pneumonia attacks, an elevated serum creatinine level, proteinuria, and high anion gap partially compensated metabolic acidosis were incidentally discovered during one of his hospitalizations. A biopsy was performed, and the results supported the diagnosis of Alport syndrome. However, a subsequent genetic test suggests the presence of MSS. Aside from NPHP, RP and CSE tested positive. Based on the fact that MSS is not a common cause of end-stage renal disease (ESRD) in pediatrics, physicians should bear in mind genetic testing as a decisive tool.

In this context, we highlighted a case of an accidentally discovered impaired renal function from first presentation to final diagnosis, with a valuable comparison with previously published similar cases.

## Introduction

Mainzer-Saldino syndrome (MSS) or conorenal syndrome (CRS) is a rare autosomal recessive ciliopathy characterized by multiorgan affection, including chronic renal disease mainly in the form of nephronophthisis (NPHP), retinitis pigmentosa (RP), cone-shaped epiphysis (CSE) and other skeletal anomalies [1,2]. Seldom cases showed other organ involvement including hepatic fibrosis, cerebellar ataxia, and short stature [2].

Multiple levels of severity have been described starting from an isolated organ affection to more severe lethal forms with multiorgan involvement. This wide presentation is due to the broad presence of cilia in different cell types, therefore a multisystemic affection is present [3].

It is primarily affined to the pathogenic genetic variants in the ciliary genes IFT140, IFT172, or IFT144, which encode elements of the intraflagellar transport protein (IFT) regulating cilium emergence, maintenance, disassembly, and material trafficking [3]. IFT140 pathogenic variation is not only confined to MSS patients, but it can also affect individuals with solitary RP, cranioectodermal dysplasia (CED), Jeune syndrome, and Opitz trigonocephaly syndrome (OTCS) [2].

Herein, we discuss a case of a child diagnosed with MSS after being misdiagnosed with Alport syndrome with over five years of follow-up.

## Case presentation

A 20-month-old male patient presented with severe pneumonia, leading to hospitalization. During this period, elevated serum creatinine, proteinuria, and partially compensated high anion gap metabolic acidosis were accidentally discovered, prompting a renal biopsy to exclude interstitial nephritis, which revealed under the electron microscope irregular thinning and thickening of the basement membrane with fibrillated texture, with a scalloped outer aspect of the glomerular capillary wall (Table 1). Podocyte's foot processes are partially fused with vacuolization and detached free in the Bowman’s space. The cell organelles in the Bowman’s space contain microvilli, which were consistent with Alport syndrome. Despite the diagnosis, no visual or auditory impairments were observed.

**Table 1:** Initial laboratory investigations. PCO_2_: the partial pressure of carbon dioxide; PO_2_: the partial pressure of oxygen; Na^+^: sodium; K^+^: potassium; HCO_3_: bicarbonate

Test	Result	Reference range
Arterial blood gases		
pH	7.318	7.35-7.45
PCO_2_	33.6 mmHg	32-48 mmHg
PO_2_	71.6 mmHg	83-108 mmHg
Electrolytes		
Na^+^	138.2 mmol/L	136-145 mmol/L
K^+^	3 mmol/L	3.5-5.1 mmol/L
Cl^-^	100 mmol/L	96-106 mmol/L
HCO_3_	16.9 mEq/L	22-26 mEq/L
Kidney function test		
Serum creatinine	1.1 mg/dL	0.2-0.5 mg/dL

The patient has a positive family history, with one sibling’s death from pneumonia at the age of six years, but there is no available data to prove if it is a part of a syndrome or not. Furthermore, there is no family history of hematuria or kidney disease. The parents are consanguineous. Pregnancy and child growth were unremarkable.

Two years later, the patient began regular hemodialysis sessions due to progressing renal failure, reaching end-stage renal disease (ESRD). On this occasion, the patient reported a history of six hospital admissions, including three for pneumonia, one with an infected line, another with a clotted line, and the last with hyperkalemia.

**Figure 1:**
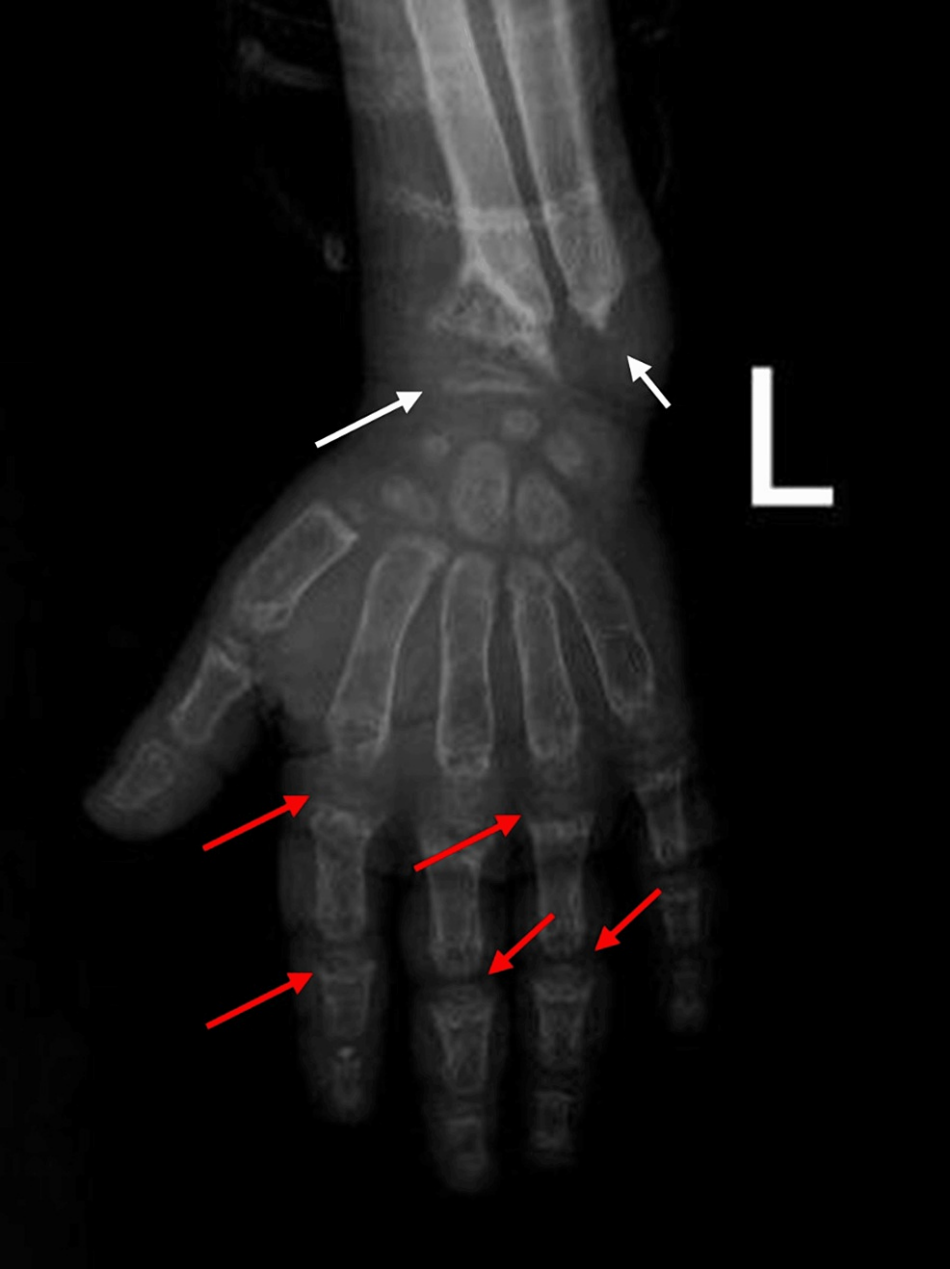
X-ray of the left hand AP view revealed diffuse osteopenia of all scanned bone coping with renal osteodystrophy. Cone-shaped phalangeal epiphysis marked by red arrow. Non-visualized ulnar epiphysis and cone-shaped radial epiphysis marked by white marrow. Widened frayed both radial and ulnar metaphysis. AP: anteroposterior

After two years of ESRD, the patient underwent whole exome sequencing (WES) to gain more clarity about his condition as part of the kidney transplantation requirements. The homozygous pathogenic variant was detected in the IFT140 gene (IFTI40:NM 014714.4:c.634G>A:p.Gly212Arg: rs201188361). It was reported that a homozygous pathogenic mutation in the IFT140 gene is related to autosomal recessive MSS. Furthermore, a hand x-ray and fundoscopic examination were performed to reveal the existence of CSE and RP, respectively (Figure 1). The results were positive. So, all the MSS triad components are present. On the contrary, physical examination of the patient showed no ataxia or short stature.

After a year, the patient faced another admission due to pneumonia. The patient was noticed to be in respiratory distress grade four and hypervolemic. Therefore, he underwent an urgent hemodialysis session, aiming to achieve the targeted weight.

**Figure 2:**
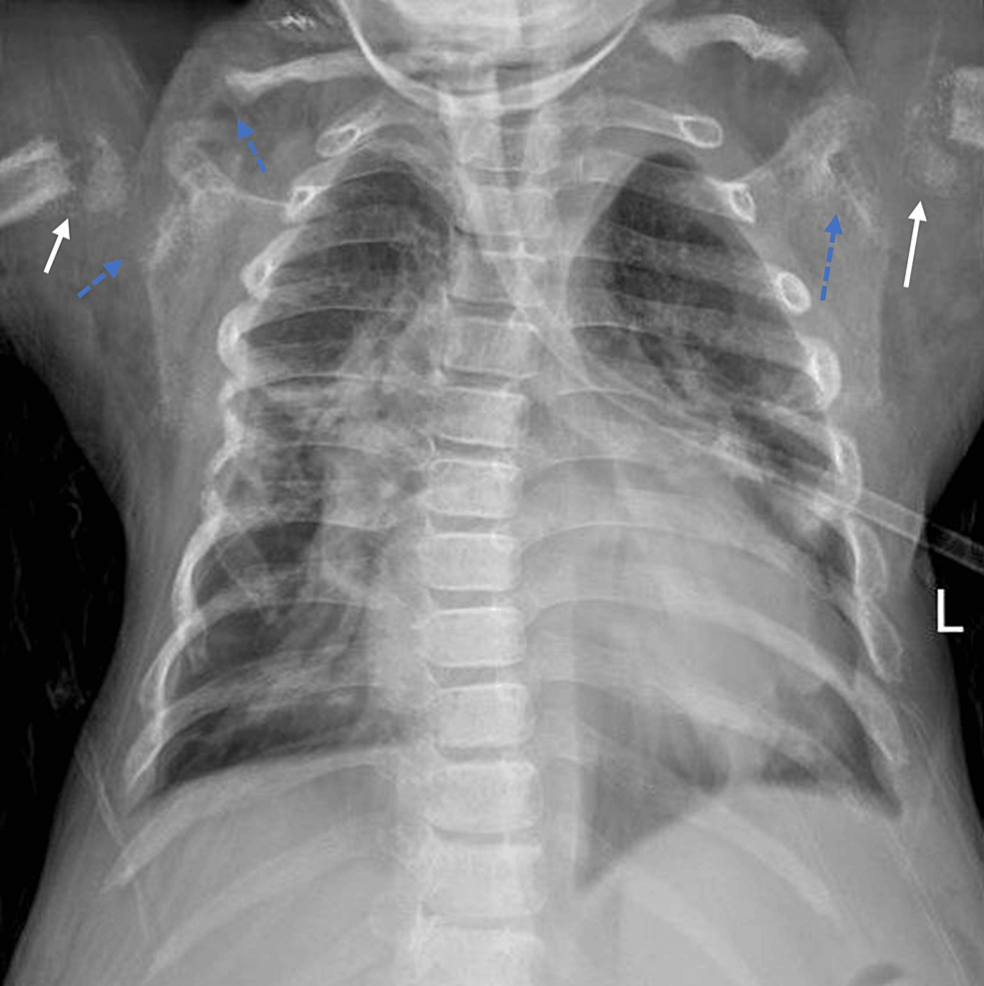
Chest x-ray PA view revealed narrow chest right middle and lower zone consolidation corresponds to recurrent pneumonia. Small irregularities in both the humeral head and the absence of osseous connection between the head and shaft, marked by the white arrow, correspond to focal proximal humeral deficiency. Subchondral and subligamentous erosions of the distal clavicle end and glenoid are marked by dashed arrows, consistent with renal osteodystrophy. PA: posteroanterior

Laboratory investigations showed normocytic normochromic anemia, leukopenia, and high serum creatinine (Table 2). The blood culture was positive for staphylococci coagulase. The other results were normal. The patient received vancomycin, levofloxacin, and azithromycin, and a nebulizer was used. Chest x-ray revealed evidence of recurrent pneumonia (Figure 2). Currently, the patient awaits kidney transplantation.

**Table 2:** Follow-up laboratory investigations.

Test	Result	Reference range
Complete blood count		
White blood cell count	3.8 K/μL	4.1-10.9 K/μL
Red blood cell count	2.67 m/μL	4.2-6.3 m/μL
Hemoglobin	8.24 g/dL	12-18 g/dL
Mean corpuscular volume	91.3 fL	80-97 fL
Mean corpuscular hemoglobin	30.9 pg	26-32 pg
Mean corpuscular hemoglobin concentration	33.9 g/dL	31-36 g/dL
Platelets	218 K/μL	140-440 K/μL
Neutrophils	50.5%	37-92%
Kidney function test		
Serum creatinine	4.4 mg/dL	0.3-0.7 mg/dL

## Discussion

Building upon the previously analyzed 16 cases of MSS reviewed in a study conducted in 2017 [4], we have encountered an additional seven cases of a confirmed MSS diagnosis published between 2017 and 2023 [2,3,5-8]. These cases did not exhibit any significant differences in symptoms or genetic background compared to the earlier cases. Eventually, most of these patients showed CSE, juvenile NPHP, and early-onset severe retinal dystrophy, indicating the necessity of these features to diagnose MSS. Based upon our literature review, less than 30 cases of this syndrome have been published till now.

Our patient presented at the age of 20 months with an accidentally discovered elevated creatinine level and proteinuria during hospitalization for pneumonia. Initially, we thought that he had Alport syndrome, as suggested by the renal biopsy. However, at the age of four years, genetic analysis revealed a homozygous pathogenic variant in IFT140 that was consistent with the diagnosis of MSS which is one of the rarest diseases.

RP is the most common ocular manifestation of MSS between the ages of two and 30 years of life [3]. Moreover, all family members with IFT140 genetic mutations had early-onset severe retinal disease between birth and four years of age [9]. Besides, CSE is one of the most eminent features of MSS [1,4,9,10].

Chest infections, which is the initial presentation of our case, are an expected event in MSS patients due to the presence of chest deformities and ciliopathies which are risk factors for chest infection [11,12]. However, chest infections in such patients are usually not severe and do not cause asphyxia [11].

ESRD can present early in the course of MSS [1,3,4]. For instance, NPHP is clinically presented in three following types: infantile, juvenile, and adolescent NPHP. Infantile NPHP usually progresses to ESRD before four years of age, while juvenile NPHP is likely to have ESRD at a median age of 13 years. Finally, adolescent NPHP can cause ESRD with 19 years mean age [13]. The prognosis of MSS is crucially related to kidney function. The mortality rate of MSS was initially approaching 50% because of renal insufficiency. Recently, the prognosis substantially improved because of mitigated treatments and kidney transplantation [10]. Due to the paucity of reported cases undergoing kidney transplantation, we can not conclude the prognosis following kidney transplantation. However, we noticed a good prognosis after kidney transplantation as experienced five years, eight years, and 30 years of survival after transplantation in some cases [1,7,10].

The diagnosis of MSS is a real challenge due to many factors. First of all, multiple syndromes present with a combination of components similar to those of MSS, so they could be misdiagnosed. For instance, Jeune asphyxiating thoracic dystrophy syndrome is a combination of both CSE and kidney disease. Moreover, Senior-Loken syndrome mostly presents with renal disease and RP [1]. Secondly, the role of ciliopathy in MSS makes the definitive diagnosis more challengeable, as it could be organ-related, as in polycystic kidney disease and NPHP, or syndromic like Jeune syndrome, Bardet-Biedl syndrome, and MSS [2]. Thirdly, IFT140 mutations are not conclusive for MSS since it is widely present in other syndromes rather than MSS. Despite this, sporadic MSS is a possibility [2,10].

## Conclusions

In conclusion, the rarity of this syndrome and the wide range of clinical and radiographic findings give genetic testing the upper hand in the definitive diagnosis conjointly with the clinical sense. It is an indispensable tool to differentiate between MSS and other analogous syndromes, especially in atypical presentations like the present case.
